# Modelling the cardiovascular system for assessing the blood pressure curve

**DOI:** 10.1186/s40929-017-0011-1

**Published:** 2017-04-04

**Authors:** T. G. Myers, Vicent Ribas Ripoll, Anna Sáez de Tejada Cuenca, Sarah L. Mitchell, Mark J. McGuinness

**Affiliations:** 1 Centre de Recerca Matemàtica, Campus de Bellaterra, Edifici C, 08193 Bellaterra, Barcelona, Spain; 2 MACSI, Department of Mathematics and Statistics, University of Limerick, Limerick, Ireland; 3 School of Mathematics, Statistics and Operations Research, Victoria University of Wellington, Wellington, New Zealand

**Keywords:** Blood pressure, Compartment model, Parameter estimation

## Abstract

A four compartment model of the cardiovascular system is developed. To allow for easy interpretation and to minimise the number of parameters, an effort was made to keep the model as simple as possible. Using a standard method (Matlab function *fminsearch*) to calculate the parameter values led to unacceptable run times or non-convergence. Consequently we developed an algorithm which first finds the most important model parameters and uses these as a basis for a four stage process which accurately determines all parameter values. This process is then applied to data from three ICU patients. Good agreement between the model and measured arterial pressure is demonstrated in all cases.

## Introduction

The human cardiovascular system conveys nutrients and oxygen to tissues and maintains the gas and fluid exchange with tissue that is necessary for homeostasis. The health of this system is important, and diagnosing cardiovascular problems in a timely and non-invasive manner is a matter of significant medical interest. A key indicator of cardiovascular health is the blood pressure and the way that it varies with time.

The term blood pressure refers to the force per unit area that blood exerts on the walls of blood vessels. This force changes both in time and with the effective distance from the aortic arch. Systolic pressure is the highest pressure, observed during ventricular contraction, whilst diastolic pressure is the lowest/baseline pressure reached during ventricular relaxation (diastole) [12]. The blood pressure can be used to identify a number of medical conditions, such as

1. Detection of cardiac arrhythmia (irregular heartbeat);
2. Estimating cardiac output;
3. Estimating hypovolemia (a decreased volume of circulating blood in the body);
4. Monitoring respiratory variation (which in turn may be related to fluid responsiveness in ventilated patients with circulatory failure);
5. Detecting attenuation of the peripheral pulse waveform — depending on different base pathologies such as sepsis or severe respiratory distress, such attenuation/damping may be considered an important measure for the assessment of microcirculation and tissue perfusion.

More comprehensive lists of examples of clinical uses for a detailed blood pressure curve may be found in [5, 18, 22, 23].

Currently there exist two principal methods for measuring blood pressure: the sphygmomanometer and the catheter. However, there is much interest in developing a reliable non-invasive, continuous monitoring technique for blood pressure. The work in this paper is motivated by a recent development which allows the blood pressure to be related to the output of a pulse oximeter (the photoplethysmograph or pleth) [19]. To maximise the information gained from this signal requires an understanding of the blood pressure curve. The specific goal of this paper is then to produce a mathematical model capable of accurately reproducing the dominant features of the blood pressure curve and in particular the dicrotic notch (a small dip in the blood pressure curve associated with aortic valve closure), and the variation due to respiratory sinus arrhythmia (RSA). Abnormalities with the dicrotic notch may indicate problems with the valve or poor vascular resistance caused, for example, by severe septic shock. To allow for easy interpretation and to minimise the number of parameters, an effort was made to keep the model as simple as possible. In the following section we will describe the compartment approach that was used to model the human cardiovascular system. Subsequent sections deal with model refinements and parameter estimation. Finally we compare our model results with data taken from a radial artery catheter. All catheters were zeroed, calibrated and a flush test performed before taking any measurement [20].

## Methods

### Mathematical models

We model the cardiovascular system using a compartment model. The main components of the system are the heart, arteries and veins. The system includes the pulmonary circulation, a closed loop through the lungs where blood is oxygenated, and the systemic circulation. Oxygenated blood enters the systemic system at the left heart and is then pumped into the aorta. The aorta branches into smaller arteries, arterioles and capillaries, where oxygen exchange takes place, and enters the systemic veins through which it flows in vessels of progressively increasing size toward the right heart. The right heart pumps CO_2_ rich blood into the lungs, closing the loop.

Compartment models are a particular type of lumped parameters model. They consist of splitting the system into sections, named compartments. The main assumption is that each compartment is homogeneous in some way. The first description of the cardiovascular system as a compartment model was published in 1733 and termed the windkessel model. This was later translated into a mathematical model in 1899, see [21]. Since the development of the windkessel model, many adaptations and different approaches have been proposed to describe the cardiovascular system. In the following we will use a differential equation approach. Differential equation models of the cardiovascular system vary from a pair of first order ordinary differential equations [15] to systems of more than 40 coupled differential-delay equations [6, 9, 25, 26]. Ottesen’s approach [15] is to split the system into arterial and venous compartments, with non-pulsatile flow, and a source term that models the effect of the left ventricle. At the other end of the spectrum, Ursino et al. and Grodins [9, 25, 26] describe the pulsatile flow of blood through multiple compartments including the lungs, compliant arteries and veins and driven by a heart that is regulated by a sophisticated nervous feedback control system that responds to blood pressure and chemistry in a variety of ways. Despite this level of complexity, in general, compartment models are conceptually quite simple. They involve dividing the cardiovascular system into a number of compliant zones or compartments and as the blood passes through each zone it must be conserved. The change in volume in each zone is simply the difference between the flux entering from upstream and that leaving downstream. The heart drives this flux and the flow is resisted by the vessels through the shear stress at vessel walls.

In the present study we have tried to take the simplest approach possible, along the lines of Ottesen’s model, whilst still being able to capture important features of the blood pressure signal. Initially we employed a three compartment model, involving the arteries, veins and left ventricle. However, one of our main goals was to have our model exhibit a dicrotic notch in arterial pressure. The dicrotic notch is the name given to the pressure fluctuation seen when the aortic valve closes. This closure is caused by the pressure drop across the valve becoming negative. The size and location in time of the notch provides important information about the health of the valve and the aorta. For example, in aortic insufficiency, the aortic valve does not completely close so that after each heart beat, the blood that has left the aorta comes back immediately to the left ventricle. This behaviour results in a complete absence of dicrotic notch in the pressure signal [10].

In the three-compartment model the arterial pressure is an average across the whole arterial system, so that using this as a measure of when the valve closes leads to late closure in the model, and a temporarily reversed blood flow throughout the arterial system.

To improve this behaviour, we introduced a new compartment to describe the exit region close to the valve. Mathematically speaking, there is no problem in dividing the cardiovascular system into any number of compartments, as is done with finite element computations. Nevertheless, to give a physical meaning to this new compartment one could think of it as representing the aortic arch. Our basic model is therefore described by the following four-compartment system

1 $$\begin{array}{@{}rcl@{}} \dot{V}_{e} &=& Q_{LV}-Q_{e} \quad \dot{V}_{a} = Q_{e}-Q_{a} \quad \dot{V}_{v} = Q_{a}-Q_{v} \quad \dot{V}_{LV} = Q_{v}-Q_{LV} ~, \end{array} $$

where *V* represents the volume and *Q* the flux of blood. The subscripts *e*,*a*,*v*,*L* *V* represent exit, arterial, venous and left ventricle and dots indicate the derivative with respect to time. The first equation indicates that the rate of change of volume in the exit region depends on the difference between the rates at which fluid flows in from the left ventricle and fluid flows out of the aortic arch. In the arteries the volume increases due to fluid flowing in from the arch and decreases as it flows out of the arteries into the veins, leading to the second equation. The third equation expresses a similar material balance for the venous system, and the fourth equation for the left ventricle. Hence these equations express conservation of blood volume. Note, we assume the blood to be incompressible, see [17], changes in pressure are associated with the compliance of blood vessels rather than the relatively small compressibility of blood itself, and conservation of blood volume is equivalent to the more fundamental principle of conservation of blood mass.

In a compliant elastic vessel we may relate the pressure to the volume via *V*=*V*_0_+*C* *p*, where *C* is a constant, termed the compliance, and *V*_0_ is a constant giving the volume at pressure *p*=0 (the value of the compliance is discussed in detail later) [12]. This equation serves to define the compliance. Note, this definition admits the possibility of zero volume, this would indicate a collapse of the vessel. Physically this is possible, however, we do not work in this regime and so assume *V*>0.

Special attention is paid to the left ventricle, where the pumping of the heart is driven by changes in the elastance (the reciprocal of the compliance, in “Comparison with experimental data” section we verify this, showing *E*≈1/*C*). Consequently, in the left ventricle we write *V*=*V*_0_+*p*/*E*_*LV*_. This equation provides a definition of elastance as the change in pressure divided by the change in volume. Differentiating the compliance and elastance definitions, we can relate volume and pressure as

2 $$\begin{array}{@{}rcl@{}} \dot{V}_{e}=C_{e} \dot{p}_{e}\qquad \dot{V}_{a}=C_{a} \dot{p}_{a} \qquad \dot{V}_{v}=C_{v} \dot{p}_{v} \qquad \dot{V}_{LV}=\frac{d}{dt} \left(\frac{p_{LV}}{E_{LV}}\right) \,. \end{array} $$

Note, we assume that the compliance of the blood vessels is a constant whereas the elastance, representing the contraction of the heart muscle, is a controlled variable that varies in a prescribed manner with time [24].

We may relate the fluxes to the pressure by considering standard, uni-directional pressure-driven laminar flow (Poiseuille flow) in a pipe which leads to a relation of the form *Q*∝*Δ* *p*. This may be expressed as *Q*=*Δ* *p*/*R* where *R* can be thought of as the effective resistance to flow [12]. Obviously the cardiovascular system does not consist of a single straight pipe and blood flow is often turbulent so this definition of *Q* is rather approximate and the resistance *R* must represent the many intricacies of the system, rather than simply the viscous resistance from the classical Poiseuille flow model.

With the fluxes written in terms of the pressure drop our initial system of differential equations may now be expressed as

3 $$\begin{array}{@{}rcl@{}} C_{e} \dot{p}_{e} &=& \frac{p_{LV}-p_{e}}{R_{e}}-\frac{p_{e}-p_{a}}{R_{a}} \end{array} $$

4 $$\begin{array}{@{}rcl@{}} C_{a} \dot{p}_{a} &=& \frac{p_{e}-p_{a}}{R_{a}}-\frac{p_{a}-p_{v}}{R_{v}} \end{array} $$

5 $$\begin{array}{@{}rcl@{}} C_{v} \dot{p}_{v} &=& \frac{p_{a}-p_{v}}{R_{v}}-\frac{p_{v}-p_{LV}}{R_{LV}} \end{array} $$

6 $$\begin{array}{@{}rcl@{}} \frac{d}{dt}\left(\frac{p_{LV}}{E_{LV}}\right) &=& \frac{p_{v}-p_{LV}}{R_{LV}}-\frac{p_{LV}-p_{e}}{R_{e}}~. \end{array} $$

#### Model refinements

The above system, Eqs. (3–6), constitutes our basic set of equations but still requires certain refinement: the driving mechanism for the flow is not defined, neither is there a mechanism to describe the dicrotic notch or the aortic valve.

The driving mechanism for the flow comes through the definition of the elastance, which serves as a simple model of the pumping action of the heart muscle. Modelling of the elastance is discussed in a number of papers. Whilst there is some difference in the fine detail, the general form is of a sequence of roughly Gaussian curves when contraction occurs, separated by flat regions denoting the relaxation [7, 14, 16]. In [6] the elastance has an approximately square wave form. However Suga [24] points out that a more gradual rise in elastance is essential if it is to be consistent with the Fenn effect in cardiac muscle, where the shortening of muscle produces heat.

The elastance also commonly exhibits a longer term variation which is one of the main causes of Respiratory Sinus Arrhythmia (RSA) [3]. RSA is a term for observed changes in heart rate associated with respiration. Heart rate is usually observed to increase during inspiration, and decrease during expiration. This is primarily due to a coupling through the vagal nervous system, and to a lesser extent is also due to the effect of breathing on the pressure in the chest cavity near the heart and to changes in peripheral tone. Denervated (transplanted) human hearts do still exhibit RSA but at around 7.9% of normal levels [2]. Dat [7] models the elastance as

7 $$\begin{array}{@{}rcl@{}} E_{LV}=E_{d}+a(t) (E_{s}-E_{d}) \end{array} $$

where the diastolic elastance *E*_*d*_ is constant and *a*(*t*)= sin2(*ω* *t*).

We employ the same form as Dat but with two important refinements. Firstly, *a*(*t*) should switch off for about 2/3 of the heart cycle [25]. If *T* represents the period of one heart-beat, then we want the *a*(*t*) term to rise from zero and fall back to zero once as *t* varies from a starting value *t*_0_ to *t*_0_+*ϕ* *T*, where *ϕ*≈1/3. So we set *ω*=2*π*/*T* and define *T*_*sys*_=*ϕ* *T*,*T*_*dia*_=(1−*ϕ*)*T*,

$$a(t)=\left\{ \begin{array}{lll} \sin^{2}\left(3 \omega(t- t_{0})/2\right) &, & t -t_{0}\in (0, T_{sys}) \\ 0 &, & t -t_{0} \in (T_{sys}, T)~. \end{array}\right. $$

The period *T* is set to be time dependent, to model RSA with heart rate varying with respiration, by choosing

8 $$\begin{array}{@{}rcl@{}} \omega=\omega_{0}+c_{3} \sin \left(\frac{\omega_{0} t}{c_{2}}\right) \,. \end{array} $$

Secondly, the peak in the elastance height also varies over the longer time-scale associated with RSA. To account for this we take the systolic elastance

9 $$\begin{array}{@{}rcl@{}} E_{s}=E_{s0}+c_{1} \sin \left(\frac{\omega_{0} t}{c_{2}}\right) \,. \end{array} $$

The constants in the above definitions *c*_1_,*c*_2_,*c*_3_ represent half the variation of elastance height, the number of heart beats per respiration (typically around 5) and half the variation of *ω* respectively. The constant *ω*_0_ is an angular frequency *ω*_0_=(2*π*/60)×*H* *R* where *H* *R* is an average heart rate in beats/minute.

The resistances *R*_*a*_,*R*_*LV*_,*R*_*v*_ are constant while *R*_*e*_ accounts for the aortic valve, that closes when the pressure drop becomes negative. Consequently *R*_*e*_ must be time-dependent. Since closure depends on the pressure difference we write

10 $$\begin{array}{@{}rcl@{}} R_{e}=R_{e0}\left[1+\epsilon_{1}(\exp(-A_{1}(p_{LV}-p_{e}))\right] \end{array} $$

where *R*_*e*0_ is the constant value when the valve is fully open. The factor *ε*_1_≪1 ensures that the exponential term remains small whenever *p*_*LV*_−*p*_*e*_>0 but it increases rapidly when *p*_*LV*_−*p*_*e*_<0. The constant *A*_1_ is chosen such that the product *A*_1_(*p*_*LV*_−*p*_*e*_) rises sufficiently rapidly as the valve closes. In practice we set $\epsilon _{1}=10^{-5}, A_{1}=\frac {1}{2}$: these values are simply chosen to provide the correct properties. Since the exit region is significantly shorter than the arterial region we also assume *R*_*e*0_≪*R*_*a*_. Another option would be to simply set a switch via a Heaviside function. However, our subsequent numerical calculations showed that this led to a poor representation of the pressure around the dicrotic notch. Ellwein et al. [8] employ a similar, but cut-off, exponential representation for all heart valves. They do not include the factor *ε*_1_≪1 but choose *A*_1_=2 which is greater than our value, and this has a similar effect.

Note, we could equally well define a valve at the entrance to the ventricle through

11 $$\begin{array}{@{}rcl@{}} R_{v}=R_{v0}\left[1+\epsilon_{2}(\exp (-A_{2}(p_{v}-p_{LV}))\right] \,. \end{array} $$

However, since this region is of lesser interest to avoid more parameter estimation we use a Heaviside function for *R*_*v*_ (tests confirm this makes no noticeable difference to the results).

The modelling of the dicrotic notch is based on the assumption that it is caused when blood attempting to flow back through the valve, due to a negative pressure difference and inertia, closes the valve and causes stretching and recoil in the valve and supporting tissues, so that blood rebounds into the exit region. Details of momentum build-up and recoil may be found in [11]. We approximate this impulse as a Gaussian, with a strength related to the pressure difference *p*_*e*_−*p*_*LV*_. Since pressure is a function of time we can represent the impulse by the following function

12 $$\begin{array}{@{}rcl@{}} f(t)=c_{4} \exp\left(-c_{5} (t-t_{n}-c_{6}\Delta t)^{2}/(\Delta t)^{2}\right) \,. \end{array} $$

The constants *c*_4_,*c*_5_,*c*_6_ indicate the height of the pulse, the sharpness and the position of the centre. The times *t*_*n*_,*n*=1,2,…, are when *p*_*e*_=*p*_*LV*_ and so indicate when the valve should begin closing. The maximum value of *f* occurs when *t*=*t*_*n*_+*Δ* *t* hence *Δ* *t* denotes the delay in closure after the pressure drop becomes negative. It therefore controls the position of the dicrotic notch and is an important indicator of the health of the valve. In general its maximum value is much lower than the other terms in the equation and so it represents only a small contribution to the pressure. In the numerical solution we use *Δ* *t* from the previous cycle, with the first value chosen as some typical value.

In the equations the function *f*(*t*) represents the flux back through the valve before it closes. It therefore represents an added flux to the exit region. This flux is lost by the left ventricle. This requires a slight modification to the governing equations. The full system to model the pressure is now given by Eqs. (4, 5) and

13 $$\begin{array}{@{}rcl@{}} C_{e} \dot{p}_{e} &=& \frac{p_{LV}-p_{e}}{R_{e}(t)}- \frac{p_{e}-p_{a}}{R_{a}} + f(t) \end{array} $$

14 $$\begin{array}{@{}rcl@{}} \frac{d}{dt} \left(\frac{p_{LV}}{E_{LV}(t)}\right) &=& \frac{p_{v}-p_{LV}}{R_{v}}-\frac{p_{LV}-p_{e}}{R_{e}(t)} -f(t) ~, \end{array} $$

with *E*_*LV*_ defined by (7) and *R*_*e*_ by (10).

## Parameter value estimation

Key to the success of the mathematical model is the choice of parameter values. Obviously this is not a simple task given that there are twenty-one parameter values as well as four initial conditions. Hence we will now describe in detail our procedure for calculating parameter values.

Our model requires parameter values appropriate for specific patients. In the following the data is taken from ICU patients who may be far from the standard values quoted in the literature (such as those provided in Table 1) and we validate the model output against arterial pressure measurements. In our initial attempts to determine parameter values we employed the Matlab function *fminsearch*, which is based on the Nelder-Mead simplex algorithm. However, this could run for days without finding a converged solution. Consequently we refined our method, in the manner detailed below, to determine a better starting point for *fminsearch*, which not only significantly improved the run time but led to more converged solutions. Our algorithm begins with a sensitivity analysis to determine which parameters are the most important (in the sense of having the greatest effect on the model’s output), these are then used in a four-stage algorithm to determine model parameter values that fit the data more accurately.

**Table 1:** Typical parameter values taken from [6, 7, 15]

Parameter	Value	Units	Parameter	Value	Units
*C*_*e*_	1.5	ml/mmHg	*C*_*a*_	1.5	ml/mmHg
*C*_*v*_	50	ml/mmHg	*R*_*e*0_	0.016	s ·mmHg/ml
*R*_*a*_	0.06	s ·mmHg/ml	*R*_*LV*_	1.2	s ·mmHg/ml
*R*_*v*_	0.016	s ·mmHg/ml	*T*_*sys*_	*T*/3	s
*T*_*dia*_	2*T*/3	s	*T*	0.9	s
*E*_*d*_	0.06	mmHg/ml	*E*_*s*0_	3.0	mmHg/ml
*ε*	10^−5^		*A*_1_	0.5	
*R*_*eM*_	10	s ·mmHg/ml	*c*_1_	0.1	mmHg/ml
*c*_2_	6	beats/breath	*c*_3_	0.01	s ^−1^
*c*_4_	500		*c*_5_	4 log100	
*c*_6_	7.5		*ω*_0_	7.54	

We will work with data sets for three patients. Details of how these data sets were obtained is provided in [20]. A number of parameter values may be roughly estimated from a given pressure signal. For example rearranging the volume pressure relation discussed in “Mathematical models” section we find

15 $$\begin{array}{@{}rcl@{}} C=\frac{V-V_{0}}{p} \,. \end{array} $$

This may be interpreted as the stroke volume (SV) to mean average pressure, see [12]. According to [1], it may be interpreted as the ratio of stroke volume to arterial pulse pressure. Since we have data for the arterial pressure we choose for an initial guess

16 $$\begin{array}{@{}rcl@{}} C_{a}=\frac{SV}{P_{a,sys}-P_{a,dia}} \,. \end{array} $$

We then set *C*_*e*_=*C*_*a*_ and *C*_*v*_=20*C*_*a*_. Note that the value of 20 assumed here for the ratio of venous to arterial compliance is consistent with a number of other studies, including [10] who cite a ratio of 8, [12] who cite a ratio of 24, [25] who uses 33, and a range of 10–20 in [13]. Similarly, the systemic resistance may be estimated from the arterial pressure signal, see [1]. Typical values for other parameter values are given the literature, see [6, 7, 15] for example.

### Sensitivity analysis

The sensitivity analysis consisted in perturbing each parameter of the model and integrating the system of equations to see how the resulting arterial pressure changes with such perturbations. This approach is based on the procedure described in [4].

**Fig. 1:**
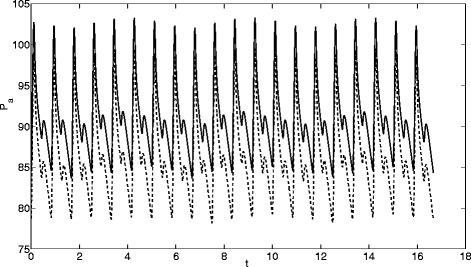
Signals for a perturbation 0.1 of *R*_*LV*_: *solid lines* *R*_*LV*_=1.1*R*_*LV*0_, *dashed lines* *R*_*LV*_=0.9*R*_*LV*0_

For each parameter *θ*, with nominal value *θ*_0_, and for a given perturbation *q*, the system was integrated for *θ*=(1−*q*)*θ*_0_ and *θ*=(1+*q*)*θ*_0_, while all the other parameters were set to their respective nominal values. Let *p*_*a*_((1−*q*)*θ*_0_) and *p*_*a*_((1+*q*)*θ*_0_) be the output arterial pressures for a perturbed parameter *θ*. Five values of the perturbation *q* were used: 0.01, 0.05, 0.1, 0.2 and 0.5. The perturbation *q*=0.01 led to very small changes in the output signal, the perturbation *q*=0.5 led to chaotic behaviour for some of the parameters. In consequence, these two values were dismissed and only the other three were used for the analysis. In Fig. 1 we present two pressure signals, *p*_*a*_((1−*q*)*θ*_0_) and *p*_*a*_((1+*q*)*θ*_0_), where the perturbed parameter was *θ*=*R*_*LV*_ and the perturbation value was *q*=0.1. From this we can see that varying the value of *R*_*LV*_ by a small amount makes significant differences to the pressure signal.

In the following we will measure the influence of the parameters through the *L*_2_ distance between both signals,

17 $$ \delta_{2}=\left(\int_{0}^{20T}{\left(p_{a}\left((1+q)\theta_{0}\right)-p_{a}\left((1-q)\theta_{0}\right)\right)^{2}} dt \right)^{\frac{1}{2}}\,.  $$

The measure was taken over a signal window of 20 beats, sufficiently far from the initial condition that the signal had settled down. In Table 2 we present the *L*_2_ distances for each parameter and the three perturbations, *q*_0_=0.05,0.1,0.2. To reduce the calculations we note that there are a number of parameters that are approximately related, *C*_*v*_=1/*E*_*d*_,*C*_*a*_=*C*_*e*_=1/*E*_*s*0_,*R*_*v*_=*R*_*e*0_ and *p*_*e*_(0)=*p*_*LV*_(0)=*p*_*a*_(0)=*p*_*a*0_. Of these only the parameter with the largest *L*_2_ distance is shown in the table. In the thesis [21] two other measures are used: the integrated absolute difference of each signal’s mean value and the integrated absolute difference of each signal’s standard deviation. These give a very similar ordering to that found by the *L*_2_ distance.

**Table 2:** *L*_2_ distance between arterial pressure signals for each parameter and perturbation

*q*=0.05		*q*=0.1		*q*=0.2	
*E*_*d*_	22.66	*E*_*d*_	32.26	*E*_*d*_	39.52
*p*_*a*0_	20.27	*p*_*a*0_	28.73	*p*_*a*0_	34.90
*ϕ*	19.75	*ϕ*	20.35	*E*_*s*0_	28.41
*ω*_0_	10.10	*E*_*s*0_	20.01	*A*	20.23
*E*_*s*0_	14.17	*ω*_0_	17.75	*ω*_0_	20.10
*R*_*LV*_	9.92	*R*_*LV*_	14.04	*R*_*LV*_	20.04
*A*	9.69	*A*	13.81	*ϕ*	19.66
*R*_*e*0_	7.09	*R*_*e*0_	9.93	*R*_*e*0_	14.11
*c*_2_	6.19	*p*_*v*0_	7.23	*p*_*v*0_	10.20
*p*_*v*0_	5.15	*c*_6_	6.51	*R*_*a*_	9.16
*c*_6_	4.78	*R*_*a*_	6.49	*c*_6_	8.60
*R*_*a*_	4.62	*c*_4_	5.88	*c*_4_	8.35
*c*_4_	4.16	*c*_2_	5.37	*c*_2_	6.81
*c*_3_	3.18	*c*_5_	4.16	*c*_5_	5.94
*ε*	3.17	*ε*	4.13	*ε*	5.76
*c*_5_	2.94	*c*_3_	3.64	*R*_*eM*_	4.70
*R*_*eM*_	2.23	*R*_*eM*_	3.51	*c*_3_	4.32
*c*_1_	1.28	*c*_1_	1.81	*c*_1_	2.56
*Δ* *t*_0_	0	*Δ* *t*_0_	0	*Δ* *t*_0_	0

The parameters have been listed in decreasing order of importance, for each degree of perturbation *q*

From Table 2 and the results described in [21] we deduce the 6 most important parameters to be: 1. *E*_*d*_ (and *C*_*v*_); 2. *E*_*s*0_ (and *C*_*a*_,*C*_*e*_); 3. *p*_*a*_(0) (and *p*_*e*_(0),*p*_*LV*_(0)); 4. *A*; 5. *R*_*LV*_; 6. *ω*_0_. The final parameter *ω*_0_ is not estimated, since it may be calculated directly from the pressure signal (and similarly for *ϕ*). Consequently in the following section we begin by estimating the remaining top five parameters and subsequently make adjustments to the remaining parameter values.

### Parameter estimation

The four stage method used to determine parameter values is described in detail in [21] so we only provide a brief outline below.

In Table 1 we present the set of parameter values used at the beginning of each calculation. The majority of these values are taken from [6, 7, 15] although some, such as *ε* are simply educated guesses. A number of parameter values are read from the data, such as *p*_*a*0_,*p*_*v*0_,*ϕ*,*Δ* *t*_0_,*ω*_0_ and so these are not included in the table.

#### Stage 1: Approximate Gradient Descent method for the five significant parameters

We begin by using a Gradient Descent method to find better estimates for the five most important parameters. If *p*_*a*_=*p*_*a*_(*t*) is the arterial pressure signal obtained by integration of the model with a certain set of parameters and $\hat {p}_{a}=\hat {p}_{a}(t)$ the arterial pressure signal recorded from a patient then we look to minimize two objective functions involving the mean and standard deviation of the signal:

18 $$\begin{array}{@{}rcl@{}} \delta_{\mu}=\left|\mu(p_{a})-\mu(\hat{p_{a}})\right|\qquad \qquad \delta_{\sigma}=\left|\sigma(p_{a})-\sigma(\hat{p_{a}})\right| \,. \end{array} $$

The Gradient Descent method with an objective function *F*(*x*) and an initial vector **x**_0_ involves iterating as follows:

19 $$ \mathbf{x}_{n+1}=\mathbf{x}_{n}-\alpha\nabla{}F(\mathbf{x}_{n}) \,,  $$

where we take the step-size *α*=0.001. For the current problem there is no explicit expression for the objective function and so we use an approximate method to determine the gradient with respect to each unknown parameter. Taking *C*_*v*_ as an example, we define *C*_*v*1_=1.1·50,*C*_*v*2_=0.9·50 (where *C*_*v*_=50 is the value quoted in Table 1). The derivative of *μ* with respect to *C*_*v*_ is approximated by

20 $$ \frac{\partial\mu}{\partial{}C_{v}}\approx\frac{\mu\left(\left.p_{a}\right|_{C_{v}=C_{v1}}\right)-\mu\left(\left.p_{a}\right|_{C_{v}=C_{v2}}\right) }{C_{v1}-C_{v2}} \,.  $$

This process may be repeated for each parameter and for the corresponding *σ* expression to obtain two gradient vectors ∇*μ*,∇*σ*. We choose the vector of parameters *Θ*=(*C*_*v*_,*p*_*a*0_,*C*_*a*_,*R*_*LV*_,*A*) with initial value *Θ*_0_=(50,35,15,1.2,0.5) and then iterate as follows:

- Set *Θ*=*Θ*_0_ and compute *ω*_0_ and *c*_2_ from the patient’s signal.
- Compute *δ*_*μ*_ and *δ*_*σ*_ and then:
  - If $\frac {\delta _{\mu }}{\mu (\hat {p}_{a})}>\frac {\delta _{\sigma }}{\sigma (\hat {p}_{a})}, \Theta _{n+1}=\Theta _{n}-\alpha \nabla \mu $
  - else *Θ*_*n*+1_=*Θ*_*n*_−*α*∇*σ*.
- Repeat until the desired tolerance is repeated.

**Fig. 2:**
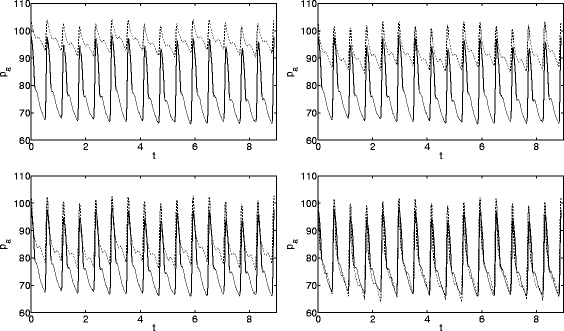
Convergence of pressure signals using the Gradient Descent method. *Solid lines* come from data, *dashed lines* from the model

Note, we use a normalised measure, e.g. *δ*(*μ*)/*μ*, in the decision process since *δ*_*μ*_ is generally greater than *δ*_*σ*_. This algorithm is used to improve the estimates for the 5 most important parameters, but it also changes the values of the dependent parameters (so in total 10 are updated). In some cases the calculation did not converge, indicating a poor initial guess however, when convergence was achieved it was typically within 20 iterations. The process is illustrated in Fig. 2. The four figures show the 5th, 9th, 13th and 17th iterations. Clearly as the process proceeds the two signals converge, until by the 17th iteration it is clear that the parameter values result in an arterial pressure curve very close to that of the patient.

#### Stage 2: Nelder-Mead method applied to the five significant parameters

The Gradient Descent method was used to refine the values of the five significant parameters from the values quoted in the literature. We now further refine their values using the Matlab function *fminsearch* which employs the Nelder-Mead method. In this case the objective function was the *L*_2_ distance between the model’s output and the recorded arterial pressure,

21 $$ \delta_{2}=\left(\int{(p_{a}-\hat{p}_{a})^{2}} dt\right)^{\frac{1}{2}} \,.  $$

As with the previous two objective functions, this was evaluated over a time period of three respiratory cycles. The initial vector of parameter values were those obtained in the previous stage. The dependent parameters were also re-calculated at each step.

#### Stage 3: Nelder-Mead method applied to all parameters

The method adopted in the previous section was then applied to all parameter values. If stages 1 and 2 are neglected then this approach does not converge. Now that the signals are relatively close, convergence is almost always achieved (this will be discussed later). From now on we neglect the interdependence of some parameters. For example, from the governing equations, Eqs. (3–6), it is clear that *E*_*d*_ can be interpreted as an inverse compliance. Hence until now we used *E*_*d*_=1/*C*_*v*_ to model venous return. We will discuss the validity of this relation in “Comparison with experimental data” section.

At the end of this stage the correspondence between *p*_*a*_ and $\hat {p}_{a}$ is in general very good with the exception of the dicrotic notch, which in the simulation occurs well below the true position. This is most likely due to minimising the *L*_2_ distance: since the notch takes up only a very small part of the signal the *L*_2_ distance will necessarily be small and so we cannot expect the same level of accuracy to be achieved in modelling the notch as in other parts of the signal.

#### Stage 4: Manual refinement

Given that the model output at this stage is so close to the signal it was decided not to build a new set of objective functions to focus solely on the minor parameters. To be specific the parameters adjusted at this stage were *c*_4_,*c*_5_,*c*_6_ (which describe the notch) and *c*_1_ (which describes the change in elastance height due to respiration). Consequently, for this final refinement we adjusted one parameter at a time up and down by about 1%.

## Results and discussion

### Comparison with experimental data

The model results are now compared against three arterial pressure signals. Direct measurements of arterial pressure were taken under a prospective clinical study conducted at the Critical Care Department and the Post-Anesthesia Care Unit of Vall d’Hebron University Hospital in Barcelona (Spain) from January 2010 to March 2012. The study was approved by the clinical research ethics committee [reference PR(AG)74/2010]. The need for informed consent was waived. All patients had mechanical ventilation. Further information on the data gathering and patient information may be found in [20]. In the thesis [21] the pressure signals of the current study and a further six cases are analysed. The patients were categorised as follows:

- **Patient 1** This patient shows normal values of blood pressure (120/70 mmHg) and heart rate (79 bpm) but a very small dicrotic notch with no real peak. Respiratory variations are small. The patient’s pathology was tagged under the category “transplantation”.
- **Patient 2** This patient’s pathology was tagged under the category “haemorrhagic”. The loss of blood led to low values of blood pressure (85/45 mmHg). Due to this fact they were administered norepinephrine (a vasoconstrictor drug). The heart rate was high (100 bpm), as a natural reaction to the low blood pressure. The dicrotic notch is absent and there is little respiratory variation in systolic pressure.
- **Patient 3** This patient shows normal values of blood pressure (135/60 mmHg) and normal heart rate (80 bpm) with a large dicrotic notch. Respiratory variations are in the order of 5 mmHg in systolic pressure. The patient’s pathology was tagged under the category “neurologic” (related to the nervous system). There was also a small dose of vasoconstrictor drugs.

**Fig. 3:**
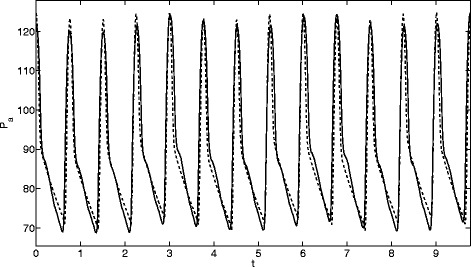
Comparison of model (*dashed line*) and measured (*solid line*) arterial pressure (mmHg) for Patient 1

**Fig. 4:**
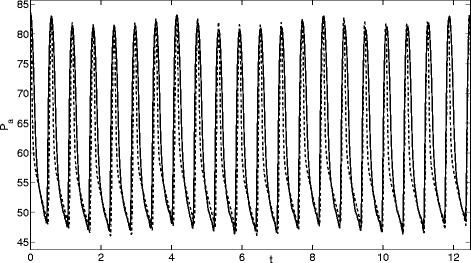
Comparison of model (*dashed line*) and measured (*solid line*) and measured arterial pressure (mmHg) for Patient 2

**Fig. 5:**
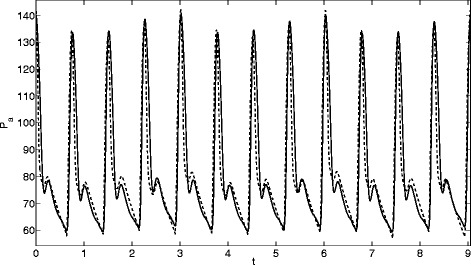
Comparison of model (*dashed line*) and measured (*solid line*) and measured arterial pressure (mmHg) for Patient 3

Comparisons of the model output against the arterial pressure for these three patients are displayed in Figs. 3, 4 and 5. The three sets of results show a wide variety of behaviour: high and low pressures, normal and fast heart rates. In Fig. 3 the dicrotic notch, which is the start of the shoulder to the right of the main peak, is very small, in Fig. 4 it is while Fig. 5 shows a significant notch. In each case the model replicates the pressure signal very accurately. However, in [21] one case could not be modelled, this was a patient with extremely high blood pressure values (220/85 mmHg) and high heart rate (104 bpm) (labelled Patient 7 in the thesis).

Tables 3, 4 and 5 show the calculated parameter values. It is quite clear that the values in these tables differ significantly from those taken from the literature and provided in Table 1. For example in Table 1 the values *C*_*v*_=59,*E*_*s*0_=3 are quoted, the corresponding values from Table 3 are 15 and 0.57. However, this may not be so unusual given that the current data set is taken from patients in the ICU, whereas the data of Table 1 presumably is not. We can also ascertain whether the assumed relations between parameters were justified. The first relation quoted was *C*_*v*_=1/*E*_*d*_: from Patient 1 we see 1/*E*_*d*_=15.26,*C*_*v*_=15. The values of *p*_*e*_(0),*p*_*LV*_(0) are very close to *p*_*a*0_ while *R*_*v*_≈*R*_*e*0_. Hence, whilst the relationships are not exact they do seem to hold approximately true in general.

**Table 3:** Parameter values for Patient 1

Parameter	Value	Parameter	Value	Parameter	Value
*R*_*e*0_	0.0055222	*p*_*a*0_	55.0000	*E*_*s*0_	0.57000
*R*_*a*_	0.020797	*p*_*v*0_	16.628	*ϕ*	0.33334
*R*_*LV*_	0.41279	*p*_*LV*0_	49.714	*R*_*eM*_	10.009
*R*_*v*_	0.0055259	*ω*_0_	8.3706	*ε*	0.00001
*C*_*e*_	1.6946	*c*_3_	0.00049874	*A*	0.40341
*C*_*a*_	2.0000	*c*_2_	4.0153	*Δ* *t*_0_	0.75786
*C*_*v*_	15.0000	*c*_1_	0.01000	*c*_4_	150.00
*p*_*e*0_	49.896	*E*_*d*_	0.065511	*c*_5_	18.445
*c*_6_	1.0000

**Table 4:** Parameter values for Patient 2

Parameter	Value	Parameter	Value	Parameter	Value
*R*_*e*0_	0.0038808	*p*_*a*0_	17.355	*E*_*s*0_	0.34
*R*_*a*_	0.0154	*p*_*v*0_	5.7844	*ϕ*	0.33273
*R*_*LV*_	0.29025	*p*_*LV*0_	17.355	*R*_*eM*_	10.116
*R*_*v*_	0.0038895	*ω*_0_	10.578	*ε*	0.000010049
*C*_*e*_	2.9196	*c*_3_	0.00010007	*A*	0.4515
*C*_*a*_	2.9193	*c*_2_	7.003	*Δ* *t*_0_	0.59617
*C*_*v*_	55.456	*c*_1_	0.006	*c*_4_	0.0000077731
*p*_*e*0_	17.355	*E*_*d*_	0.0181	*c*_5_	18.436
*c*_6_	4.5053

**Table 5:** Parameter values for Patient 3

Parameter	Value	Parameter	Value	Parameter	Value
*R*_*e*0_	0.0049252	*p*_*a*0_	21.781	*E*_*s*0_	0.64627
*R*_*a*_	0.018469	*p*_*v*0_	7.2597	*ϕ*	0.33338
*R*_*LV*_	0.39929	*p*_*LV*0_	21.761	*R*_*eM*_	10.004
*R*_*v*_	0.0049253	*ω*_0_	8.3339	*ε*	0.000010007
*C*_*e*_	1.54	*c*_3_	0.0001251	*A*	0.43995
*C*_*a*_	1.0459	*c*_2_	4.0001	*Δ* *t*_0_	0.75182
*C*_*v*_	50.000	*c*_1_	0.015	*c*_4_	400
*p*_*e*0_	21.783	*E*_*d*_	0.029022	*c*_5_	1.000
*c*_6_	3.5000				

## Conclusions

In this paper we put forward a simple model of the blood pressure curve. The model takes into account the dicrotic notch or incissura that results from the closure of the aortic valve. This is important because it allows our model to account for aortic insufficiency [10] and pressure changes in the vascular tree that result from changes in vascular compliance and vascular resistance. Another contribution of the work is the simple refinement to model respiratory sinus arrythmia in the blood pressure curves.

One important limitation of this model is that it cannot account for differences in blood volume. For example, in the event of haemorraghic shock blood volume becomes variable and the variations observed in pulse pressure can no longer be attributed to respiratory sinus arrythmia. However, the simplicity of the model allows for further refinements in order to model different conditions including blood loss and haemorragic shock.

Beyond the simplicity of the model and the parameters obtained for different patients, it is also worth mentioning that further studies are needed to assess the physiological and clinical relevance of the parameters that we obtain. The parameters obtained in this paper are physiologically relevant but it is also necessary to assess their value for different conditions and a much larger number of patients.

The results presented in the previous sections clearly indicate that our four-compartment model can accurately reproduce a blood pressure signal, with the correct choice of parameter values. The model is capable of reproducing a wide variety of pressure signals. In fact, if the goal is simply a rough characterisation of the blood pressure signal, then the initial stages of the analysis proved sufficient. This showed that the 5 most important parameters in our model are *E*_*d*_,*E*_*s*0_,*p*_*a*_(0),*A*,*R*_*LV*_. Provided these are determined accurately, in our case this was achieved through the gradient descent method, then the model will provide a satisfactory description of the signal.

The four-stage parameter estimation method was vital to the success of the analysis. We have made little effort to optimise this process, since our main goal was to develop and verify the compartment model. However, this provides one direction for future work. For example the final stage involved manual refinement since the *L*_2_ error for the dicrotic notch parameters was small due to the small width of the region. The technique of previous stages could instead be applied to a reduced region around the notch. Employing the Conjugate Gradient method rather than simple Gradient Descent should reduce the number of iterations required.

Based on the output of our model in the future we hope to be able to interpret the parameter values in some way in order to make some recommendation concerning the health of the patient. This will comprise the next stage of this investigation. In order to achieve this we note that the vascular tree can be modelled as a Non-Linear Time-Variant Channel and there are tools that can either linearize this system or we can simply live with these non-linearities and make predictions (Kalman Filters). On the positive side we have a large amount of data from arterial catheters on which to validate the method.
